# Levels, Exposure and Risk Assessment to Metals in Blood of Adult Working Population in Valencia Region (Spain)

**DOI:** 10.3390/toxics14100874

**Published:** 2026-09-30

**Authors:** Carmen Sáez, Alfredo Sánchez, Pablo Dualde, Miguel Ángel Aguirre, Antonio Canals, Marta Esteban-López, Ana Cañas-Portilla, Susana Pedraza-Díaz, Clara Coscollà

**Affiliations:** 1 Department of Analytical Chemistry, Nutrition and Food Science and University Institute of Materials, University of Alicante, 03690 San Vicente del Raspeig, Spain; 2 Public Health Laboratory of Alicante, 6 Plaza de España, 03010 Alicante, Spain; 3 Foundation for the Promotion of Health and Biomedical Research in the Valencian Region, FISABIO-Public Health, 21 Avenida Catalunya, 46020 Valencia, Spain; 4 National Centre for Environmental Health, Instituto de Salud Carlos III, Ctra. Majadahonda–Pozuelo, Km.2, 28220 Majadahonda, Spain

**Keywords:** metals, human biomonitoring, risk assessment, adults, blood

## Abstract

Exposure of the general population to essential and toxic metals represents an important public health concern due to their accumulation in the body and their potential adverse effects on multiple target organs. This study, conducted within the framework of the BIOMOVAL project, determined whole-blood concentrations of 19 metals (As, Ba, Be, Cd, Co, Cs, Cu, Hg, Mn, Mo, Ni, Pb, Pt, Sb, Se, Tl, U, V, and Zn) in 541 working adults recruited during 2021 in the Valencian Community, Spain. Furthermore, data on sociodemographic characteristics, occupational history, dietary habits, and lifestyle factors were also collected. Essential metals, including Cu, Co, Zn, Mn, and Se, were detected with 100% detection rates. Regarding toxic metals, the concentration of Pb was higher in male subjects and smokers. For Hg, the hazard quotient (HQ) at the 95th percentile was 3.28, and 41% of the population exceeded the HBM-I reference value, which may indicate potential health concerns for a substantial proportion of the study population. Cd, although safe on average (HQ = 0.17), exceeded the threshold at the 95th percentile (HQ = 1.04). Zn, on the other hand, had an HQ of 1.23 at the 95th percentile, and 23% of population exceeded the selected Biomonitoring Equivalent value. This study highlights the importance of implementing strategies to reduce exposure to some metals, as well as sustaining human biomonitoring programmes to support evidence-based public health policies and protect the population.

## 1. Introduction

### 1.1. Essential and Toxic Metals

Human exposure to metals, both essential and toxic, is an increasingly serious public health concern due to the potential effects of these compounds on human physiology and health [1,2]. Essential metals, including copper (Cu) [3], zinc (Zn) [4], manganese (Mn) [5], selenium (Se) [6], cobalt (Co) [7], molybdenum (Mo) [8], and vanadium (V) [9], are essential for biochemical processes and cellular function, acting as cofactors for enzymes and antioxidant compounds [10,11,12]. However, elevated levels of these metals can be harmful, causing toxic effects and altering metabolism which increases the likelihood of developing degenerative diseases, cardiovascular diseases or cancer [13,14]. Toxic metals, on the other hand, such as cadmium (Cd) [15], lead (Pb) [16], mercury (Hg) [17], arsenic (As) [18], nickel (Ni) [19], thallium (Tl) [20], barium (Ba) [21], caesium (Cs) [22], antimony (Sb) [23], beryllium (Be) [24], uranium (U) [25], and platinum (Pt) [26], have no known biologically beneficial function and instead may pose adverse health effects due to their persistence and bioaccumulative properties and impact target organs, such as the liver, kidneys, central nervous system, and cardiovascular system [27,28,29]. Due to their severe toxicity and widespread environmental presence, a number of these metals, particularly Cd, Pb, Hg, As, Ni, and Sb are listed as priority chemicals by international organisations [30].

### 1.2. Sources of Exposure

The sources of metal exposure are diverse, including ingestion of contaminated food and water, inhalation of particulate matter from industrial and urban areas, and specific lifestyle factors [2,31,32]. The regional geography of a given area, the methods used to process foodstuffs, and certain occupations can have a significant impact on exposure levels [33,34]. Furthermore, habits such as smoking, and the use of household products may serve to increase the uptake of metals [35,36,37,38,39,40]. Mercury is a highly toxic metal of major public health concern. Human exposure occurs predominantly through the consumption of aquatic food products, including marine fish, freshwater fish, and seafood. Lead is another toxic metal of major public health concern. Human exposure to lead has traditionally been linked to sources such as leaded gasoline, mining activities, and battery recycling; however, less obvious sources, including wall paints and materials used in water distribution systems, may also contribute to exposure [2]. Arsenic exposure is commonly associated with contaminated drinking water, crops irrigated with arsenic-contaminated water, and tobacco use [41]. In addition, cigarette smoking represents an important source of exposure to several toxic metals, particularly cadmium (Cd) [42].

### 1.3. Human Biomonitoring

Given the ubiquitous nature of these sources, human biomonitoring (HBM) has become an essential tool for assessing exposure in the general population and generating data for comprehensive risk assessments [43,44]. Human biomonitoring involves the analysis of biological samples, including blood, serum, urine, and breast milk, allowing direct estimation of the body’s metal burden [45,46]. This tool is of great importance for the assessment of nutritional status regarding essential metals as well as the identification of population subgroups that are at an elevated risk from toxic metals as a result of chronic or elevated exposure, as well as to track the exposure trends and investigate how sociodemographic and lifestyle factors, such as age, gender, socioeconomic status, and diet, influence metal levels [33,34].

Furthermore, HBM offers crucial data for public health risk assessment, enabling the levels identified to be benchmarked against reference values established by health agencies [43]. This analysis allows the identification of population groups at risk, thereby facilitating the implementation of regulatory policies and intervention strategies designed to reduce exposure [47]. Moreover, these data are useful for developing public knowledge campaigns to encourage lifestyles that minimise the absorption of toxic metals, thus improving quality of life and preventing chronic diseases.

### 1.4. Objectives

This study forms part of the BIOMOVAL project, was conducted with the support of FISABIO and the Public Health Department of the Generalitat Valenciana. It aimed to assess the exposure to various essential and toxic metals in a sample of 541 individuals selected as representative of the adult working population of the Valencian Region. In addition to measuring metal concentrations, a comprehensive survey was conducted to collect socio-demographic and lifestyle data, allowing for a detailed exploration of potential associations between metal concentrations and exposure factors. Furthermore, a risk assessment was conducted, thereby providing a robust evidence base for formulating public health policies and managing metal exposure in the adult working population. To the authors’ knowledge, this is the first study to assess these metals in a population of working adults from the Valencia Region.

## 2. Materials and Methods

### 2.1. Study Participants and Sample Collection

The current study forms part of the BIOMOVAL project, supported by the Foundation for the Promotion of Health and Biomedical Research in the Valencian Region (FISABIO) and the General Directorate of Public Health and Addictions.

Participants were recruited in 2021 at a number of Prevention Health Centres affiliated with Unimat Prevention, the University of Jaume I (UJI) and the Association of Occupational Risk Prevention Services of the Valencian Community (SERPRECOVA). The recruitment process has already been explained in detail by Peris-Camarasa [48]. In brief, the recruitment process followed the recommendations of the HBM4EU initiative, which suggests including at least 500 individuals per age group to ensure representativeness and statistical robustness in HBM [49]. Participants were selected through occupational health mutual insurance centres; therefore, the study population was limited to employed adults up to 65 years of age. Stratification was carried out by age, sex, province, and economic sector, by census data [50] to ensure proportional representativeness from the three provinces of the Valencia Region: Castellón (63), Alicante (205), and Valencia (280).

A total of 548 volunteers between the ages of 18 and 65 were recruited. Venous blood was collected in a series of green-capped, metal-free tubes filled with heparin anticoagulant BD Vacutainer^®^ (BD, Plymouth, UK). To ensure sample homogeneity, the tubes were inverted eight times after collection. They were then quickly transported using isothermal packaging within twenty-four hours of collection. To prevent chemical degradation, the packaging ensured refrigerated transport at 4 °C until the samples were stored in 10 mL aliquots at −80 °C at the Biobank for Biomedical Research and Public Health of the Valencian Community (IBSPCV Biobank, Spain; PT13/0010/0064).

The study was approved by the Ethics Committee for Research of the General Directorate of Public Health and Research of the Foundation for the Promotion of Health and Biomedical Research of the Valencian Region. Before data and sample collection, all participants provided written informed consent. All personal data were handled, transferred, and analysed in a pseudonymised way and will be retained only for the necessary processing period. The processing of data adhered to all national and EU legal and ethical guidelines, specifically the General Data Protection Regulation.

### 2.2. Sample Analysis

As, Ba, Be, Cd, Co, Cs, Cu, Hg Mn, Mo, Ni, Pb, Pt, Sb, Se, Tl, U, V, and Zn concentrations were measured in 541 whole blood samples out of the 548 participants, as some participants did not provide a sufficient sample volume for analysis. For the determination of all analytes, except Hg, samples were diluted 1:20 (*v*/*v*). For this purpose, a 0.5 mL sample was made up to a final volume of 10 mL with 50 μL of HNO_3_ (69%), 50 μL of Triton 0.1% and deionised water. The resulting solution was vortex-mixed for subsequent determination using an inductively coupled plasma mass spectrometer (ICP-MS). The chemical analysis of the metals determined in the present study was performed at the Public Health Laboratory of Alicante, Spain, using an iCAP RQ ICP-MS (Thermo Scientific, Waltham, MA, USA). Table S1 shows the isotopes measured and internal standards used for these metals. Operating conditions are shown in Table S2. The ICP-MS method utilized helium collision mode (Kinetic Energy Discrimination, KED) for all elements to minimize spectral interferences and enhance analytical precision. Further information regarding the analytical sequence, routine analytical procedures, and interferences is available on Tables S3 and S4.

Quality control samples were included in each analytical batch. Five different quality control samples were chosen to monitor the analytical sequence: Initial Calibration Verification (ICV), Initial Calibration Blank (ICB), reagent blanks, Certified Reference Materials (CRM) Seronorm™—Trace Elements Whole Blood Level I and II (Billingstad, Norway), and Continuous Calibration Verification (CCV), as well as internal standard signal monitoring. The limits of quantification (LoQ) and results for reference materials are presented in Table S5.

For the analysis of total Hg no prior sample treatment was required. Thus, 100 mg of whole blood was weighed in a quartz boat and placed in the direct mercury analyser DMA-80 Tricell Milestone, (Sorisole, Italy). The instrument conditions are given in Table S6. The internal quality control was performed through addition–recovery studies at different concentration levels, obtaining recovery values within the range of 80–120%.

### 2.3. Population Characteristics

Table 1 presents some basic characteristics of the studied population. All participants completed a self-reporting questionnaire that included comprehensive information on the relevant characteristics. The food consumption frequencies by group were transformed into semi-quantitative intakes and subsequently converted into kilograms per month, in a manner analogous to that employed by Yusà et al. [51] in previous studies. Additionally, the group was given a 24-h reminder questionnaire to evaluate their recent metal exposure. Further details regarding participants’ dietary habits can be found in Table 2.

### 2.4. Statistical Analysis

The statistical analysis was conducted using IBM SPSS Statistics 29.0.2.0. A descriptive summary of metal concentration was performed, including detection frequency (DF, %), arithmetic mean (AM), geometric mean (GM), lowest and maximum values, standard deviation (SD), and the 25th, 50th, 75th, and 95th percentiles (P25, P50, P75, and P95). Additionally, the questionnaires were analysed to extract the most significant characteristics of the research population. Quantitative variables were averaged using their mean, minimum, and maximum values; qualitative variables were expressed using absolute frequencies and percentages (Table 1 and Table 2). Furthermore, information collected from food consumption during the previous 24 h was grouped by food categories, including fruits, vegetables, meat, fish, etc. Spearman’s correlation test was used to detect relationships between metal concentrations. Linear regression models were also constructed to investigate the connection between each metal and exposure factors. The candidate explanatory variables included the sociodemographic, lifestyle and dietary variables collected in the study. Considering the non-normal distribution of the raw data (μg/L), variables were selected using a forward stepwise procedure based on the corrected Akaike Information Criterion (AICc). In the final models, the explanatory variables of age, sex and BMI were retained following the selection process [52]. Metal concentrations were log10-transformed, and 0.05 was chosen as the level of statistical significance. Normality, non-autocorrelation, homoscedasticity, and non-multicollinearity were among the tests used to confirm the regression assumptions once the models were constructed. For this, the Durbin-Watson statistics, the Kolmogorov–Smirnov test, scatter plots, and tolerance and condition indexes were used.

### 2.5. Exposure and Risk Assessment

For risk assessment purposes, hazard quotients (HQs) for essential and toxic metals with established biomonitoring equivalent (BE) reference values (Cd, Se, Mo, and Zn) and for the toxic metal (Hg) with available HBM-I/II reference values were determined. The risk descriptor was calculated as the ratio between the biomarker concentration in the 95th percentile or GM and the chemical-specific BE or HBM-I/II value, depending on the metal, as follows:

HQ = [Biomarker, (95th percentile or GM)]/BE or HBM-I/II [53].

The BEs and HBM values applied in this study were derived from established toxicological reference values and translated into blood concentrations using pharmacokinetic modelling. For Cd, a BE of 1.4 µg/L in blood was used, based on the chronic Minimal Risk Level (MRL) established by the Agency for Toxic Substances and Disease Registry (ATSDR), as reported by Hays et al. [54]. For Se, Mo, and Zn, the BEs were derived from the Upper Intake Levels (ULs) defined by the Institute of Medicine (IOM), corresponding to blood concentrations of 480 µg/L for Se [55], 5.04 µg/L for Mo [56], and 6682 µg/L for Zn [57]. In the case of Hg, HBM-I (5 µg/L) and HBM-II (15 µg/L) values established by the German Human Biomonitoring Commission were applied [58]. These values are based on toxicological and epidemiological evidence. HBM-I represents a level above which exposure should be further assessed, while HBM-II indicates a concentration at which adverse health effects cannot be excluded.

An HQ value < 1 indicates that exposure to a given metal may not result in adverse health effects for the target population.

## 3. Results and Discussion

### 3.1. Metal Levels in Blood Samples

Table 3 shows the results obtained for all the metals analysed in the study. For the essential metals, the DF was 100% for five of the seven metals studied (i.e., Cu, Co, Mn, Se, and Zn). The DF decreases to 51% for Mo and is practically non-existent for V (<2%). In the case of toxic metals, a DF of almost 100% was obtained for Sb, As, Cs, Pb and Hg. A DF of 68% was observed for Cd, while the remaining metals exhibited a DF < 20% (i.e., Ba, Ni, Pt, Tl, and U). Additionally, Be was not detected in any sample.

Results obtained were compared with analogous studies conducted in other countries (Table 4). Particularly relevant data are available from national human biomonitoring programs, including those conducted by Health Canada [59], the National Health and Nutrition Examination Survey (NHANES) in the United States of America [60], and the China National Human Biomonitoring program [61].

As shown in Table 4, Cu and Zn concentrations were broadly comparable across all studies evaluated. In contrast, in the present study, concentrations of Mo, Co, Se, Hg, and As were generally higher than or similar to those reported in previous studies, with a few exceptions. Higher Hg and Se concentrations were reported in Brazil [38] and Greenland [62], while Co concentrations were higher in Brazil [38] and Australia [63]. In addition, As concentrations were higher in Greenland [62]. The higher concentrations of Hg, Se, and As observed in the present study may be attributable to dietary factors, particularly the consumption of fish and other marine products, as previously reported by Wielsøe et al. [62].

In contrast, Cs, Mn, and Cd concentrations in the present study were generally lower than or similar to those reported in other studies. Notable exceptions included lower Mn concentrations in Barcelona [64] and lower Cd concentrations in Brazil [38] and Canada [59]. The relatively low Cd levels observed in the present study could be related to differences in dietary habits and lifestyle factors, particularly tobacco consumption, which is known to increase Cd levels [38].

For Sb and Pb, the concentrations observed in the present study fell within the intermediate range of values reported across the studies considered.

As for the remaining metals (i.e., Ba, Be, Ni, Pt, Tl, V, and U), the DF was <20%, and only a limited number of studies reporting whole-blood concentrations of these metals were identified. In general, the concentrations reported in previous studies were below the LoQ established in the present study. However, higher levels of Ni, Tl, and Ba were reported in Brazil [38], whereas higher concentrations of Ni and Tl were observed in Australia [63].

**Table 4:** Comparative data on working adult’s metal concentrations in whole blood in other studies.

	Study	Country/Location	Year of Sampling	N	Age (Years)	AM (Arithmetic Mean)	GM (Geometric Mean)
**Essential metals**							
**Copper**							
	Present study	Spain (Valencia)	2021	541	18–65	863 µg/L	843 µg/L
	[38]	Brazil	2022	1024	18–80	1037 µg/L	
	[63]	Australia	2018	120	18–73	840 µg/L	
	[65]	China (Wuhan)	2016–2018	461	5–80	791.39 µg/L	783.76 µg/L
	[66]	Germany	2020	102	20–66	951 µg/L	934 µg/L
	[64]	Spain (Barcelona)	2016	240	19–80	-	712 µg/L
	[62]	Greenland	2019–2020	163	24–55	944 µg/L	-
**Cobalt**							
	Present study	Spain (Valencia)	2021	541	18–65	0.24 µg/L	0.22 µg/L
	[38]	Brazil	2022	1024	18–80	0.29 µg/L	-
	[61]	China	2017–2018	9945	18–80	-	0.14 µg/L
	[60]	USA	2017–2018	3520	40-		0.173 µg/L
	[63]	Australia	2018	120	18–73	0.33 µg/L	
	[66]	Germany	2020	102	20–66	0.16 µg/L	0.13 µg/L
	[67]	Serbia	2019–2020	984	18–65	0.25 µg/L	-
**Manganese**							
	Present study	Spain (Valencia)	2021	541	18–65	7.38 µg/L	7.06 µg/L
	[38]	Brazil	2022	1024	18–80	14.2 µg/L	
	[61]	China	2017–2018	9945	18–80	-	10.74 µg/L
	[60]	USA	2017–2018	5021	20-		9.28 µg/L
	[63]	Australia	2018	120	18–73	9.7 µg/L	
	[65]	China (Wuhan)	2016–2018	260	5–80	12.93 µg/L	12.40 µg/L
	[66]	Germany	2020	102	20–66	8.9 µg/L	8.5 µg/L
	[64]	Spain (Barcelona)	2016	240	19–80	-	3.87 µg/L
	[62]	Greenland	2019–2020	163	24–55	10.3 µg/L	-
**Molybdenum**							
	Present study	Spain (Valencia)	2021	541	18–65	0.67 µg/L	<LoQ
	[61]	China	2017–2018	9945	18–80	-	0.61 µg/L
	[63]	Australia	2018	120	18–73	0.49 µg/L	
	[66]	Germany	2020	102	20–66	0.49 µg/L	0.44 µg/L
	[64]	Spain (Barcelona)	2016	240	19–80	-	2.09 µg/L
**Selenium**							
	Present study	Spain (Valencia)	2021	541	18–65	173 µg/L	170 µg/L
	[38]	Brazil	2022	1024	18–80	184 µg/L	
	[61]	China	2017–2018	9945	18–80	-	95.56 µg/L
	[60]	USA	2017–2018	5021	20-		191 µg/L
	[63]	Australia	2018	120	18–73	141 µg/L	
	[66]	Germany	2020	102	20–66	107 µg/L	106 µg/L
	[59]	Canada	2018–2019	4596	3–79	170 µg/L	-
	[64]	Spain (Barcelona)	2016	240	19–80	-	181.5 µg/L
	[62]	Greenland	2019–2020	163	24–55	232 µg/L	-
**Zinc**							
	Present study	Spain (Valencia)	2021	541	18–65	5822 µg/L	5886 µg/L
	[38]	Brazil	2022	1024	18–80	6130 µg/L	
	[63]	Australia	2018	120	18–73	6750 µg/L	
	[65]	China (Wuhan)	2016–2018	461	5–80	5950 µg/L	5850 µg/L
	[66]	Germany	2020	102	20–66	5660 µg/L	5620 µg/L
	[64]	Spain (Barcelona)	2016	240	19–80	-	5904 µg/L
	[62]	Greenland	2019–2020	163	24–55	5520 µg/L	-
**Vanadium**							
	Present study	Spain (Valencia)	2021	541	18–65	<LoQ	<LoQ
	[63]	Australia	2018	120	18–73	<0.45 µg/L (LoQ)	
	[66]	Germany	2020	102	20–66	0.018 µg/L	0.017 µg/L
**Non-** **e** **ssential** **m** **etals**							
**Antimony**							
	Present study	Spain (Valencia)	2021	541	18–65	2.34 µg/L	2.18 µg/L
	[61]	China	2017–2018	9945	18–80	-	2.22 µg/L
	[63]	Australia	2018	120	18–73	4.1 µg/L	-
	[66]	Germany	2020	102	20–66	<0.07 µg/L (LoQ)	<0.07 µg/L (LoQ)
	[64]	Spain (Barcelona)	2016	240	19–80	-	3.83 µg/L
**Arsenic**							
	Present study	Spain (Valencia)	2021	541	18–65	4.74 µg/L	3.13 µg/L
	[38]	Brazil	2022	1024	18–80	4.4 µg/L	
	[61]	China	2017–2018	9945	18–80	-	1.11 µg/L
	[63]	Australia	2018	120	18–73	2.2 µg/L	
	[65]	China (Wuhan)	2016–2018	260	5–80	3.03 µg/L	2.25 µg/L
	[66]	Germany	2020	102	20–66	0.87 µg/L	0.47 µg/L
	[59]	Canada	2018–2019	2531	3–79	4.8 µg/L	-
	[64]	Spain (Barcelona)	2016	240	19–80	-	3.15 µg/L
	[62]	Greenland	2019–2020	163	24–55	8.84 µg/L	-
**Barium**							
	Present study	Spain (Valencia)	2021	541	18–65	<LoQ	<LoQ
	[38]	Brazil	2022	1024	18–80	5.5 µg/L	-
	[66]	Germany	2020	102	20–66	0.78 µg/L	0.38 µg/L
**Berilium**							
	Present study	Spain (Valencia)	2021	541	18–65	<LoQ	<LoQ
	[38]	Brazil	2022	1024	18–80	0.019 µg/L	-
	[66]	Germany	2020	102	20–66	<0.02 µg/L (LoQ)	<0.02 µg/L (LoQ)
**Cadmium**							
	Present study	Spain (Valencia)	2021	541	18–65	0.42 µg/L	0.24 µg/L
	[38]	Brazil	2022	1024	18–80	0.28 µg/L	
	[61]	China	2017–2018	9945	18–80	-	0.90 µg/L
	[60]	USA	2017–2018	5021	20-	-	0.295 µg/L
	[63]	Australia	2018	120	18–73	0.80 µg/L	-
	[65]	China (Wuhan)	2016–2018	260	5–80	1.27 µg/L	0.70 µg/L
	[66]	Germany	2020	102	20–66	0.55 µg/L	0.36 µg/L
	[59]	Canada	2018–2019	4596	3–79	0.24 µg/L	-
	[64]	Spain (Barcelona)	2016	240	19–80	-	0.315 µg/L
	[62]	Greenland	2019–2020	163	24–55	0.644 µg/L	-
**Caesium**							
	Present study	Spain (Valencia)	2021	541	18–65	2.69 µg/L	2.54 µg/L
	[38]	Brazil	2022	1024	18–80	14.1 µg/L	-
	[66]	Germany	2020	102	20–66	4.2 µg/L	3.9 µg/L
**Lead**							
	Present study	Spain (Valencia)	2021	541	18–65	14.02 µg/L	12.24 µg/L
	[38]	Brazil	2022	1024	18–80	53 µg/L	
	[61]	China	2017–2018	9945	18–80	-	21.92 µg/L
	[68]	France	2014–2016	999	18–74	-	18.5 µg/L
	[60]	USA	2017–2018	5021	20-	-	8.55 µg/L
	[63]	Australia	2018	120	18–73	13.6 µg/L	-
	[65]	China (Wuhan)	2016–2018	260	5–80	21.86 µg/L	17.84 µg/L
	[66]	Germany	2020	102	20–66	13 µg/L	11 µg/L
	[59]	Canada	2018–2019	4596	3–79	8.1 µg/L	-
	[64]	Spain (Barcelona)	2016	240	19–80	-	17.1 µg/L
	[62]	Greenland	2019–2020	163	24–55	12.1 µg/L	-
**Mercury**							
	Present study	Spain (Valencia)	2021	541	18–65	5.9 µg/L	4.4 µg/L
	[38]	Brazil	2022	1024	18–80	26.2 µg/L	-
	[61]	China	2017–2018	9945	18–80	-	0.89 µg/L
	[60]	USA	2017–2018	5021	20-	-	0.764 µg/L
	[63]	Australia	2018	120	18–73	2.0 µg/L	-
	[65]	China (Wuhan)	2016–2018	260	5–80	2.57 µg/L	1.90 µg/L
	[66]	Germany	2020	102	20–66	0.65 µg/L	0.41 µg/L
	[59]	Canada	2018–2019	4596	3–79	0.71 µg/L	-
	[64]	Spain (Barcelona)	2016	240	19–80	-	2.72 µg/L
	[62]	Greenland	2019–2020	163	24–55	6.39 µg/L	-
**Nickel**							
	Present study	Spain (Valencia)	2021	541	18–65	<LoQ	<LoQ
	[38]	Brazil	2022	1024	18–80	5.7 µg/L	-
	[61]	China	2017–2018	9945	18–80	-	0.49 µg/L
	[63]	Australia	2018	120	18–73	2.0 µg/L	-
	[66]	Germany	2020	102	20–66	0.40 µg/L	0.38 µg/L
	[67]	Serbia	2019–2020	984	18–65	0.53 µg/L	-
**Platinum**							
	Present study	Spain (Valencia)	2021	541	18–65	<LoQ	<LoQ
	[66]	Germany	2020	102	20–66	<0.1 µg/L (LoQ)	<0.1 µg/L (LoQ)
**Thalium**							
	Present study	Spain (Valencia)	2021	541	18–65	<LoQ	<LoQ
	[38]	Brazil	2022	1024	18–80	0.09 µg/L	-
	[61]	China	2017–2018	9945	18–80	-	0.03 µg/L
	[63]	Australia	2018	120	18–73	0.06 µg/L	-
	[65]	China (Wuhan)	2016–2018	176	5–80	<LoQ	<LoQ
	[66]	Germany	2020	102	20–66	<0.04 µg/L (LoQ)	<0.04 µg/L (LoQ)
	[64]	Spain (Barcelona)	2016	240	19–80	-	0.009 µg/L
**Uranium**							
	Present study	Spain (Valencia)	2021	541	18–65	<LoQ	<LoQ
	[38]	Brazil	2022	1024	18–80	0.05 µg/L	-
	[63]	Australia	2018	120	18–73	<0.1 µg/L (LoQ)	-
	[66]	Germany	2020	102	20–66	<0.002 µg/L (LoQ)	<0.002 µg/L (LoQ)

### 3.2. Predictors of Exposure to Metals

One of the main objectives of this study was to identify socio-demographic, lifestyle, and dietary characteristics that influence the levels of priority metals and other toxic and essential metals with a DF > 50%. Additionally, correlations were identified between the metals Cd, Pb, Hg, As, and Sb, as well as Mn, Mo, Co, Cu, Zn, Se and Cs. However, Ni was excluded from further investigation due to its low percentage of detection (<2%). Table 5 illustrates the characteristics that significantly influenced (*p*-value < 0.05) the levels of each metal.

#### 3.2.1. Hg

Total mercury concentrations in blood are primarily attributable to dietary exposure to organic Hg species, especially methylmercury [69]. In the present study, consumption of seafood, particularly shellfish, within the previous 24 h significantly elevated blood Hg levels. The frequency of mollusk and fish consumption was also identified as a contributing factor. This finding is corroborated by numerous previous studies [70,71,72], given the well-established knowledge that seafood contains elevated levels of Hg [73,74,75]. Furthermore, fruit consumption within the previous 24 h was also associated with higher blood Hg concentrations, which may be attributed to environmental contamination of fruits, particularly in regions with contaminated soil, air, or water [76,77]. However, the relationship between fruit consumption and Hg exposure should be interpreted cautiously, as the authors are not aware of any evidence of high Hg pollution in water, soil, or air in the study area. In addition, older participants exhibited higher blood Hg concentrations than younger participants. This finding may be related to the cumulative effect of Hg in the body, a hypothesis put forward by some researchers [78,79,80].

#### 3.2.2. As

The blood As concentrations increased in correlation with recent shellfish consumption (within the past 24 h), the frequency of shellfish consumption, and the use of sunscreen within the past 24 h. This may be related to the prevalence of As contamination in shellfish and molluscan species, similar to Hg [81,82]. Additionally, certain cosmetic products contain metals, like As, which may contribute to dermal exposure, resulting in elevated blood concentrations [83].

#### 3.2.3. Pb

The concentration of Pb in the sample increased with age. The concentration of Pb in smokers was higher due to the presence of trace amounts of this metal in tobacco, and this association has also been reported by Shakeri [84,85]. Furthermore, male subjects exhibited higher levels than females, likely attributable to occupational or metabolic differences [86]. Participants employed in the construction sector exhibited higher blood Pb concentrations due to their proximity to aged paints, solder, and other contaminated materials [87,88].

#### 3.2.4. Cd

Lower blood Cd concentrations were observed in non-smokers and former smokers, a phenomenon that may be related to smoking representing a primary source of Cd exposure [89,90,91]. A positive association was observed between age and increased blood Cd levels; however, a study conducted by Nordberg [92] did not find the same correlation. Regarding sex, males exhibited lower Cd levels than females. This difference may reflect physiological and metabolic differences, as women tend to exhibit higher Cd uptake in the presence of iron deficiency, a condition that is more prevalent in women due to menstrual losses [93,94].

#### 3.2.5. Sb

In the case of Sb, the levels observed demonstrated a positive correlation with the time spent with windows open, which may facilitate the entry of pollutant particles from the external air. Furthermore, increases were observed in individuals engaged in agricultural activities, potentially attributable to the contamination of soils [95,96].

#### 3.2.6. Cs

The concentration of Cs in blood was influenced by several demographic, dietary, and occupational factors. Regarding sex, males exhibited higher Cs levels than females. This finding may reflect variations in occupational activities and dietary patterns [97]. Furthermore, dietary habits have been identified as a contributing factor. There was a positive correlation between coffee consumption and blood Cs concentration. This may be related to the water used for its preparation; the frequency of tap water consumption was also associated with a slight increase in Cs levels as it may contain traces of Cs [98]. Similarly, the consumption of vegetables within the previous 24 h was linked to an elevated Cs concentration, likely due to the accumulation of Cs in soils and its transfer to cultivated vegetables [99,100]. According to this last association, people working in the agricultural sector were associated with elevated Cs in comparison with the service sector.

#### 3.2.7. Essential Metals

Essential metals are indispensable for optimal bodily function; however, both excess and deficiency can be harmful. One of the aims of this study was to elucidate the sociodemographic, lifestyle, and dietary factors that most influence their levels in the blood.

Beginning with Cu, the regression coefficient for male sex was negative, indicating that males tended to have lower Cu levels than females. This discrepancy could be attributed to physiological differences, as Cu is inversely related to testosterone [101]. Similar gender-related differences have been reported by Buxaderas and R. Farré-Rovira [102]. Furthermore, the intermittent utilisation of skincare products was also observed to elevate Cu levels. This is presumably attributable to indirect exposure to trace amounts of Cu in certain cosmetic products [103]. Additionally, the frequency of herbal tea consumption was observed to be associated with elevated Cu levels. This is potentially attributable to certain herbal teas naturally containing Cu, depending on the origin of the plants used in their preparation [104]. The consumption of seafood within the previous 24 h was also observed to elevate Cu levels, given that seafood is a rich source of this metal due to its natural concentration in marine organisms [105].

Zn levels observed in men were higher than in women. Those employed in the construction industry may have higher concentrations as this is one of the main sources of exposure to this metal [4]. Alcohol consumption frequency may also increase levels, and similar associations have been reported by Li et al. [106].

The present study suggested that Se levels are higher in non-smokers and former smokers than in smokers. This finding suggests that smoking may reduce the absorption or increase the excretion of this trace metal. Recent consumption of herbal teas and yoghurt was observed to increase levels, which is likely attributable to these foods being natural sources of Se [107,108].

The concentration of Mn in the blood was mainly associated with smoking status. Former smokers and non-smokers exhibited higher levels of blood Mn compared with current smokers. This association has also been reported by Uz et al. [109] and it potentially due to interactions between tobacco smoke constituents and Mn metabolism.

The use of supplements was identified as the primary variable associated with a notable elevation in blood concentrations of Mo. This is because such products frequently contain Mo as a fundamental component in their formulation, which is essential for enzyme metabolism [110]. Similarly, the consumption of yoghurt and cereals was observed to increase Mo levels, which is likely attributable to the presence of Mo in these products [111]. The consumption of ecological food had a negative effect on levels, which may be related to the lower mineral content in soil used for agricultural practices, despite the more sustainable nature of these methods.

The concentration of Co in the blood was subject to several different factors that exerted a significant influence. Concerning sex, males exhibited lower Co levels than females. Occasional lipstick use throughout the year was associated with higher Co levels compared with daily lipstick use. However, no significant differences were observed between non-users and daily users. Therefore, this association should be interpreted with caution. This association may be related to the presence of trace amounts of Co in certain cosmetics [112], the absorption of which varies according to the frequency of use. Nevertheless, the daily use of lipstick did not exhibit a notable impact on Co levels.

### 3.3. Risk Assessment

**Figure 1:**
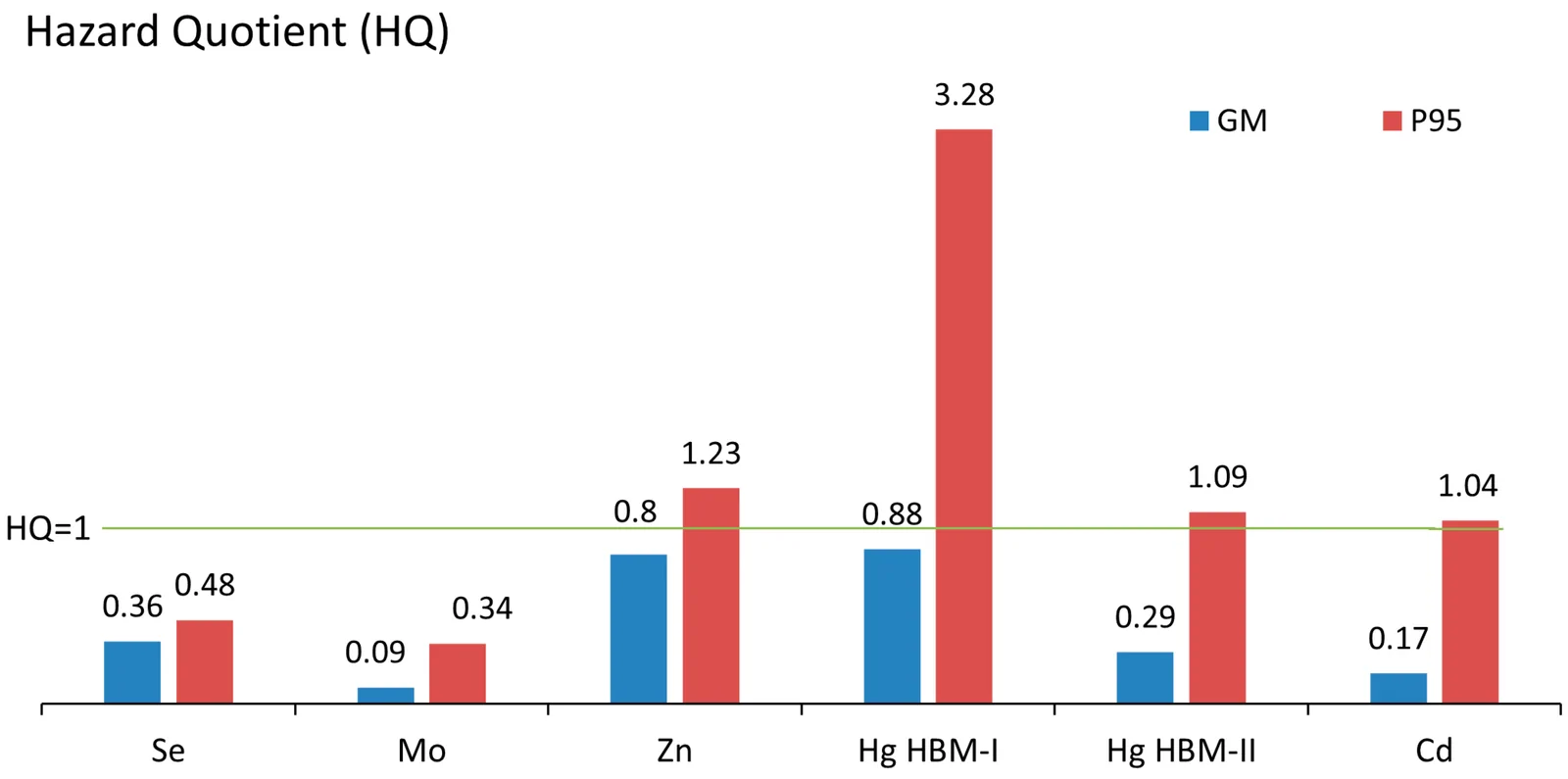
Results of HQ for Se, Mo, Zn, Hg and Cd.

Figure 1 illustrates HQs of various metals employing two metrics: GM, represented by blue bars, and P95, represented by red bars. The green horizontal line, marked ‘HQ = 1’, represents the reference threshold. An HQ value > 1 indicates a potential health risk, whereas HQ values ≤ 1 are typically considered to indicate acceptable level of risk.

The BE values applied in this study were 1.4 µg/L for Cd; 480 µg/L for Se; 5.04 µg/L for Mo, and 6682 µg/L for Zn. In the case of Hg, HBM-I (5 µg/L) and HBM-II (15 µg/L) values were used (see Section 2.5).

For Se and Mo, both the GM and P95 remain below the reference value of 1, with respective values of 0.36 and 0.48 for Se and 0.09 and 0.34 for Mo. This indicated that the potential health risk associated with these two essential metals is low in the population under analysis.

A calculation of the HQ for Zn using the GM resulted in a value of 0.85, also below the risk threshold. Nevertheless, if P95 is used, it reaches a value of 1.23, indicating that a minor proportion of the population may be exposed to Zn levels that could potentially pose a health risk. Indeed, 23% of the studied population exceeded the biological reference value (BE) for Zn. However, urinary Zn is considered a more reliable biomarker for Zn exposure than blood concentrations [57]. In a previous study conducted in the same population using urinary measurements, HQ values were below 1 at both the GM and the P95 [113]. Therefore, risk assessment results of Zn based on blood concentrations should be interpreted with caution.

For Cd, an HQ of 0.17 is obtained using the GM, which is below the reference limit. However, using P95, it is 1.04, which is also slightly above the threshold of 1, and 5% of the analyzed population exceeded the BE for this metal. This finding contrasts with the Cd risk assessment previously conducted in urine samples from the same population, in which the HQ at the 95th percentile (P95) was substantially lower (0.51) [113].

In the case of Hg, two HBM levels were assessed: Hg HBM-I and Hg HBM-II. The Hg HBM-I level represents a concentration below which no adverse health effects are anticipated; therefore, no action is required. In contrast, level HBM-II is regarded as an intervention threshold, as it denotes a concentration at which the health risk increases and exposure reduction and biomedical care have been identified as necessary [58]. Concerning Hg HBM-I, using the GM, the HQ is 0.88, while using P95, it reaches 3.28. This suggests a potential risk. Additionally, 41% of the study group had a blood Hg concentration above the recommended level. Regarding Hg HBM-II, the HQ is 0.29 when using the GM and 1.09 when using P95, which is just above the threshold of 1. Additionally, the acceptable threshold was exceeded in 6.3% of the population. Exposure to Hg is particularly concerning among women of childbearing age because methylmercury can be transferred to the fetus, and prenatal development is recognized as a highly sensitive period for Hg exposure [114]. Restricting the analysis to women aged 18–50 years yielded HQs of 0.80 (GM) and 2.98 (P95) based on the HBM-I guidance value, and 0.27 (GM) and 0.99 (P95) based on the HBM-II guidance value. These estimates were marginally lower than those observed for the overall population but remained of a similar magnitude. However, risk assessment results of Hg should be interpreted with caution since additional factors relevant to the toxicological interpretation, such as the Hg/Se molar ratio, may affect the interpretation of Hg-associated health risks.

## 4. Conclusions

The present study demonstrates the value of assessing metal exposure in the general population through human biomonitoring to identify both exposure patterns and associated risk factors. The data obtained, although specific to the adult working population of the Valencian Region, offer a valuable resource for comparison at the national and even international level, thereby contributing to the advancement of knowledge on metal exposure in human populations. The detection of 19 metals in blood, including essential metals, such as Cu, Zn, Mn, and Se, as well as toxicants, such as Hg, Pb, and Cd, suggests multiple exposures from diet, occupation, and other lifestyle factors. The levels of Hg found in a significant proportion of the population may be a cause for concern. In addition, the analysis shows that certain groups, such as construction workers and smokers, are at increased risk of exposure to some metals, which are linked to specific work activities and lifestyles.

The calculation of HQs allowed the identification of potentially affected populations. Most essential metals were found to be at acceptable levels; however, potential risks related to toxic metals were observed. These results emphasise the need to prioritise specific actions to reduce exposure, such as education on dietary habits, improvements in occupational safety, and increased monitoring of food and materials that may contain these contaminants.

**Study limitations:** Some limitations should be considered when interpreting the findings of this study. First, participants were recruited through occupational health services; therefore, the study population was restricted to actively employed adults aged 18–65 years, limiting the generalizability of the results to the wider population. Second, biological samples were collected in 2021, and exposure patterns may have changed since then. Third, whole blood is not the preferred biological matrix for assessing exposure to several metals, including As, Sb, Pt, Tl, U, V, and Zn, for which urinary biomarkers are generally considered more informative [57,115,116,117,118,119,120]. However, whole blood concentrations are also interesting and urinary concentrations of these metals in the studied population have already been reported by our group in a previous publication [113]. Fourth, no correction for multiple testing was applied in the regression analyses, increasing the possibility of type I errors. Finally, information regarding the specific fish species consumed was unavailable, limiting the identification of dietary sources contributing to elevated blood Hg concentrations.

## Figures and Tables

**Table 1:** Lifestyle, demographic factors and population characteristics (N = 541).

Variable	n (%)/Mean (Range)
*Province*	
Castellón	63 (11.6%)
Valencia	276 (51.0%)
Alicante	202 (37.3%)
*Sex*	
Male	282 (52.1%)
Female	259 (47.9%)
*Height* (cm)	170 (148–195)
*Weight* (Kg)	75 (40–199)
*BMI*	25.5 (17–56)
*Age*	42 (18–65)
*Socio-economic sector*	
Farming	10 (1.8%)
Industry	43 (7.9%)
Construction	34 (6.3%)
Services	454 (83.9%)
*Distance from Industry*	
Very far (>1000 m)	352 (65.1%)
Far (200–1000 m)	141 (26.1%)
Close (100–200 m)	27 (5.0%)
Very close (50–100 m)	21 (3.8%)
*Smoking Status*	
Smoker	144 (26.6%)
Former Smoker	165 (30.5%)
*Non-Smoker*	232 (42.9%)
Frequency of use of:	
*Pet care products ^a^*	
Never	350 (64.7%)
Several times per year	129 (23.8%)
Monthly	60 (11.1%)
Used in last 3 days:	
*Plant care products ^b^*	
Yes	11 (2.0%)
No	514 (95.0%)
*Pet care products ^c^*	
Yes	22 (4.1%)
No	505 (93.3%)
Used in last 24 h:	
*Shampoo ^d^*	
Yes	520 (96.1%)
No	20 (3.7%)
*Wipes ^e^*	
Yes	144 (26.6%)
No	392 (72.5%)
Sunscreen ^f^	
Yes	95 (17.6%)
No	442 (81.7%)

^*a*^ Missing data of frequency of use for pet care products: 2; ^*b*^ Missing data of Plant care products: 16; ^*c*^ Missing data of used in last 3 days pet care products: 14; ^*d*^ Missing data of used in last 24 h shampoo: 1; ^*e*^ Missing data of used in last 24 h wipes: 5; ^f^ Missing data of used in last 24 h sunscreen: 4.

**Table 2:** Frequency of food consumption and cooking practices, as well as the consumption of food 24 h prior to the study. (N = 541).

Variable	n (%)/Mean (Min–Max)
*Frequency of use of cooking practices*	
*Frying ^a^*	
Never or <1/month	81 (15.0%)
<1/week	281 (51.9%)
∼4/week	159 (29.4%)
>1/day	18 (3.3%)
*Baking ^b^*	
Never or <1/month	30 (5.5%)
<1/week	226 (41.8%)
∼4/week	248 (45.8%)
>1/day	35 (6.5%)
*Roasting ^c^*	
Never or <1/month	47 (8.7%)
<1/week	199 (36.8%)
∼4/week	244 (45.1%)
>1/day	50 (9.2%)
*People who used these culinary practices last 24 h*	
*Frying ^d^*	
Yes	151 (27.9%)
No	389 (71.9%)
*Baking ^e^*	
Yes	197 (36.4%)
No	341 (63.0%)
*Roasting ^f^*	
Yes	173 (32.0%)
No	362 (66.9%)
*Frequency of consumption of food groups (Kg or L/month) ^g^*	
*Fish ^h^*	1.0 (0–11)
*Shellfish*	0.20 (0–2)
*Meat ^i^*	1.4 (0–7.5)
*Vegetables*	6.28 (0–36)
*Fruits*	8.3 (0–36)
*Dairy products*	5.2 (0–22.5)
*Eggs*	1.49 (0–16)
*Cereals*	2.7 (0–13.5)
*Pre-cooked food*	0.37 (0–7.5)
*Oil*	0.5 (0–1.8)
*Bottled water*	19 (0–36)
*Alcohol*	1.91 (0–36)
*Coffee*	2.18 (0–9)
*Juices*	1.62 (0–36)
*Infusions*	1.64 (0–27)
*Frequency of consumption of these food last 24 h (Kg or L)*	
*Seafood*	0.08 (0–1.80)
*Meat*	0.16 (0–1.3)
*Vegetables*	0.27 (0–1.60)
*Fruits*	0.23 (0–1.46)
*Yogurt*	0.04 (0–0.63)
*Eggs*	0.05 (0–0.5)
*Rice*	0.02 (0–0.3)
*Cereals*	0.04 (0–0.45)
*Bread*	0.16 (0–0.8)
*Pre-cooked food*	8 (0–200) *
*Oil*	0.02 (0–0.7)
*Bottled water*	0.45 (0–5)
*Alcohol*	0.06 (0–0.4)
*Coffee*	0.02 (0–0.3)
*Juices*	0.02 (0–0.2)
*Milkshakes*	0.1 (0–1.6)
*Infusions*	0.014 (0–0.4)
*Supplements*	0.5 (0–20) *

^*a*^ Missing data of frequency of consumption of frying: 2; ^*b*^ Missing data of frequency of consumption of baking: 2; ^*c*^ Missing data of frequency of consumption of roasting: 1; ^*d*^ Missing data of used in last 24 h frying: 1; ^*e*^ Missing data of used in last 24 h baking: 3; ^*f*^ Missing data of used in last 24 h roasting: 6; ^*g*^ Semiquantification (see Table S7); ^*h*^ Missing data of frequency of consumption of fish: 1; ^*i*^ Missing data of frequency of consumption of meat: 1. * Expressed in grams.

**Table 3:** Metal concentrations in whole blood (µg/L) of working adult population (N = 541).

^a^	Hg	Be	V	Mn	Co	Ni	Cu	Zn	As	Se	Mo	Cd	Sb	Cs	Ba	Pt	Tl	Pb	U
**DF (%)**	96	0	1.3	100	100	13	100	100	97	100	51	68	99	100	16	0.9	18	100	0.7
**LoQ**	1.0	0.68	0.48	0.84	0.06	1.0	1.54	11.6	0.46	0.77	0.49	0.15	1.08	0.06	0.84	0.30	0.04	0.76	0.24
**Min.**	<LoQ	<LoQ	<LoQ	2.22	0.10	<LoQ	489	2664	<LoQ	101.3	<LoQ	<LoQ	<LoQ	0.89	<LoQ	<LoQ	<LoQ	2.50	<LoQ
**P25**	2.8	<LoQ	<LoQ	5.82	0.17	<LoQ	763	4923	1.83	151.3	<LoQ	<LoQ	1.76	2.05	<LoQ	<LoQ	<LoQ	8.58	<LoQ
**Median**	4.3	<LoQ	<LoQ	7.03	0.20	<LoQ	824	5575	3.17	168.9	0.49	0.23	2.36	2.51	<LoQ	<LoQ	<LoQ	12.3	<LoQ
**AM**	5.9	<LoQ	<LoQ	7.38	0.24	0.64	863	5822	4.74	172.9	0.67	0.42	2.34	2.69	0.69	<LoQ	<LoQ	14.0	<LoQ
**GM**	4.4	<LoQ	<LoQ	7.06	0.22	0.57	846	5686	3.13	170.5	0.47	0.24	2.18	2.54	0.53	<LoQ	<LoQ	12.2	<LoQ
**P75**	7.0	<LoQ	<LoQ	8.50	0.26	0.49	914	6607	5.75	189.9	0.81	0.46	2.75	3.15	0.42	<LoQ	<LoQ	17.2	<LoQ
**P95**	16.4	<LoQ	<LoQ	11.6	0.49	1.41	1111	8204	13.24	228.9	1.73	1.46	3.36	4.36	2.06	<LoQ	0.06	29.4	<LoQ
**Max.**	40.2	<LoQ	1.19	26.1	1.26	9.88	2419	10,298	76.14	305.9	14.0	7.36	19.7	9.05	7.88	0.74	0.49	72.8	1.30
**SD**	5.0	-	0.06	2.35	0.14	0.57	195	1285	5.95	29.25	0.92	0.59	1.09	0.97	0.85	0.04	0.03	8.03	0.06

^a^ all parameters expressed as µg/L. DF: Frequency of detection; LoQ: Limit of Quantification; Min: Minimum; P25, P75 and P95: percentiles 25th, 75th and 95th respectively; AM: arithmetic mean; GM: Geometric Mean; Max: Maximum; SD: Standard deviation.

**Table 5:** Results of the multiple linear regression models for log (metals) concentrations in whole blood in working adult population of Valencian Region (Spain).

Metals	Factors	Estimated Coefficients (95% CI)	Standard Error	*p*-Value
**Hg**	Seafood 24 h	0.231 (0.071, 0.390)	0.081	0.005
	Frequency shellfish	0.134 (0.031, 0.238)	0.053	0.011
	Frequency fish	0.032 (0.009, 0.056)	0.012	0.007
	Fruit 24 h	0.172 (0.063, 0.281)	0.055	0.002
	Age	0.004 (0.001, 0.006)	0.001	0.004
**As**	Seafood 24 h	0.457 (0.294, 0.621)	0.083	<0.0001
	Frequency shellfish	0.182 (0.079, 0.286)	0.053	0.001
	Sunscreen Frequency: Never	−0.029 (−0.156, 0.099)	0.065	0.659
	Sunscreen Frequency: Several times per year	0.095 (0.003, 0.186)	0.047	0.042
	Sunscreen Frequency: Daily	0 (reference)	N/A	N/A
	Skincare 24 h	0.162 (0.046, 0.278)	0.059	0.006
**Cd**	Smoking Status: Non-Smoker	−0.726 (−0.789, −0.662)	0.032	<0.0001
	Smoking Status: Ex-Smoker	−0.597 (−0.665, −0.529)	0.035	<0.0001
	Smoking Status: Smoker	0 (reference)	N/A	N/A
	Age	0.006 (0.004, 0.008)	0.001	<0.0001
	Gender: Male	−0.073 (−0.124, −0.022)	0.026	0.005
	Gender: Female	0 (reference)	N/A	N/A
**Pb**	Age	0.008 (0.006, 0.009)	0.001	<0.0001
	Smoking Status: Smoker	0.113 (0.077, 0.150)	0.018	<0.0001
	Smoking Status: Ex-Smoker	0.025 (−0.019, 0.070)	0.023	0.266
	Smoking Status: Non-Smoker	0 (reference)	-	-
	Sex: Male	0.117 (0.070, 0.163)	0.024	<0.0001
	Sex: Female	0 (reference)	-	-
	Socio-economy sector: Farming	−0.000 (−0.116, 0.115)	0.059	0.993
	Socio-economy sector: Industry	0.009 (−0.047, 0.066)	0.029	0.746
	Socio-economy sector: Construction	0.091 (0.027, 0.154)	0.032	0.005
	Socio-economy sector: Services	0 (reference)	-	-
**Sb**	Open window hours per day	0.002 (0.001, 0.004)	0.001	0.005
	Socio-economy sector: Farming	0.094 (0.015, 0.173)	0.040	0.020
	Socio-economy sector: Industry	−0.026 (−0.066, 0.015)	0.021	0.210
	Socio-economy sector: Construction	−0.009 (−0.053, 0.035)	0.022	0.689
	Socio-economy sector: Services	0 (reference)	-	-
**Cu**	Sex: Male	−0.058 (−0.077, −0.038)	0.010	<0.0001
	Sex: Female	0 (reference)	-	-
	Sunscreen Frequency: Never	0.006 (−0.018, 0.030)	0.012	0.610
	Sunscreen Frequency: Several times per year	−0.017 (−0.033, 0.000)	0.009	0.052
	Sunscreen Frequency: Daily	0 (reference)	-	-
	Skincare Frequency: Never	0.004 (−0.015, 0.023)	0.010	0.686
	Skincare Frequency: Several times per year	0.018 (0.004, 0.031)	0.007	0.011
	Skincare Frequency: Daily	0 (reference)	-	-
	Frequency Infusions	0.003 (0.001, 0.004)	0.001	0.006
	Seafood 24 h	0.040 (0.011, 0.069)	0.015	0.007
**Zn**	Sex: Male	0.071 (0.051, 0.091)	0.010	<0.0001
	Sex: Female	0 (reference)	-	-
	Socio-economy sector: Farming	−0.027 (−0.079, 0.026)	0.027	0.315
	Socio-economy sector: Industry	0.025 (−0.002, 0.052)	0.014	0.069
	Socio-economy sector: Construction	0.033 (0.002, 0.063)	0.015	0.034
	Socio-economy sector: Services	0 (reference)	-	-
	Frequency Alcohol	0.003 (0.001, 0.005)	0.001	0.006
**Se**	Frequency Infusions	0.004 (0.002, 0.006)	0.001	<0.0001
	Smoking Status: Non-Smoker	0.020 (0.006, 0.034)	0.007	0.004
	Smoking Status: Ex-Smoker	0.026 (0.011, 0.041)	0.008	0.001
	Smoking Status: Smoker	0 (reference)	-	-
	Yogurt 24 h	0.111 (0.037, 0.185)	0.038	0.003
**Mn**	Smoking Status: Non-Smoker	0.049 (0.025, 0.073)	0.012	<0.0001
	Smoking Status: Ex-Smoker	0.033 (0.007, 0.060)	0.013	0.13
	Smoking Status: Smoker	0 (reference)	-	-
**Mo**	Bread 24 h	0.217 (0.086, 0.348)	0.067	0.001
	Cereals 24 h	4.152 (1.350, 6.954)	1.426	0.004
	Suplements 24 h	12.542 (3.074, 22.009)	4.819	0.010
	Yogurt 24 h	0.406 (0.068, 0.745)	0.172	0.019
	Age	0.003 (0, 0.005)	0.001	0.019
	Use of Shampoo 24 h: Yes	0.157 (0.014, 0.301)	0.073	0.032
	Use of Shampoo 24 h: No	0 (reference)	-	-
**Co**	Province: Castellón	0.040 (−0.008, 0.088)	0.055	0.100
	Province: Valencia	−0.060 (−0.090, 0.029)	0.016	<0.001
	Province: Alicante	0 (reference)	-	-
	bottled water 24 h	0.028 (0.011, 0.045)	0.009	0.001
	BMI	−0.005 (−0.008, 0.001)	0.002	0.006
	Sex: Male	−0.067 (−0.117, 0.018)	0.025	0.008
	Sex: Female	0 (reference)	-	-
	Lipsticks Frequency: Never	0.047 (−0.027, 0.121)	0.038	0.210
	Lipsticks Frequency: Several times per year	0.069 (0.006, 0.132)	0.032	0.032
	Lipsticks Frequency: Daily	0 (reference)	-	-
**Cs**	Coffee Frequency	0.011 (0.005, 0.017)	0.003	0.001
	Sex: Male	0.034 (0.010, 0.058)	0.012	0.006
	Sex: Female	0 (reference)	-	-
	Socio-economy sector: Farming	0.117 (0.029, 0.205)	0.045	0.010
	Socio-economy sector: Industry	0.007 (−0.036, 0.050)	0.022	0.743
	Socio-economy sector: Construction	−0.013 (−0.062, 0.036)	0.025	0.611
	Socio-economy sector: Services	0 (reference)	-	-
	Vegetables 24 h	0.057 (0.010, 0.105)	0.024	0.019
	Frequency Tap Water	0.001 (0, 0.002)	0.001	0.022

## Data Availability

The authors state that the data supporting the study’s findings are included in the paper and in the Supplementary Information Files. If raw data in another format or presentation is required, it can be obtained from the corresponding author upon request.

## Supplementary Materials

The following supporting information can be downloaded at: https://www.mdpi.com/article/10.3390/toxics14100874/s1, Table S1: Isotopes measured and internal standard selected on ICP-MS. Table S2: ICP-MS instrumental operating conditions. Table S3: The analytical sequence followed in each batch of samples analysed. Table S4: Polyatomic interferences in ICP-MS. Table S5: LOQ and Results for the Reference materials: Seronorm™—Trace Elements Whole Blood Level I and II. Table S6: Elemental mercury analyzer operating conditions. Table S7: Semiquantification of frequency of consumption of food groups (kg or L/month) procedure.
