# Supplementary material for: Levels, Exposure and Risk Assessment to Metals in Blood of Adult Working Population in Valencia Region (Spain)
Source: Toxics. 2026 Sep 30;14(10):874. doi: 10.3390/toxics14100874 (PMC13709369; doi:10.3390/toxics14100874)
Supplement: Supplementary file 1 [file toxics-14-00874-s001.zip › toxics-4499755-supplementary.pdf]

toxics

MDPI

Article

Levels, Exposure and Risk Assessment to Metals in Blood of Adult Working Population in Valencia Region (Spain)

Carmen Sáez [1], Alfredo Sánchez [2], Pablo Dualde [3,*], Miguel Ángel Aguirre [1], Antonio Canals [1], Marta Esteban-López [4], Ana Cañas-Portilla [4], Susana Pedraza-Díaz [4] and Clara Coscollà [3]

1 Department of Analytical Chemistry, Nutrition and Food Science and University Institute of Materials, University of Alicante, 03690 San Vicente del Raspeig, Spain; carmen.saez@ua.es (C.S.); aguirre.pastor@ua.es (M.Á.A.); a.canals@ua.es (A.C.)
2 Public Health Laboratory of Alicante, 6 Plaza de España, 03010 Alicante, Spain; sanchez_alfagu@gva.es
3 Foundation for the Promotion of Health and Biomedical Research in the Valencian Region, FISABIO-Public Health, 21 Avenida Catalunya, 46020 Valencia, Spain; clara.coscolla@fisabio.es
4 National Centre for Environmental Health, Instituto de Salud Carlos III, Ctra. Majadahonda–Pozuelo, Km.2, 28220, Majadahonda, Spain; m.esteban@isciii.es (M.E.-L.); acanas@isciii.es (A.C.); spedraza@isciii.es (S.P.-D.)
* Correspondence: pablo.dualde@fisabio.es; Tel.: +34-961926323

Table S1. Isotopes measured and internal standard selected on ICP-MS.

Analyte	Mass (amu)	Internal standard
Antimony	^{121}Sb	^{103}Rh
Arsenic	^{75}As	^{128}Te
Barium	^{137}Ba	^{103}Rh
Beryllium	^{9}Be	^{45}Sc
Cadmium	^{111}Cd	^{103}Rh
Cobalt	^{59}Co	^{72}Ge
Caesium	^{133}Cs	^{103}Rh
Copper	^{63}Cu	^{103}Rh
Lead	$^{206,207,208}Pb$	^{209}Bi
Manganese	^{55}Mn	^{72}Ge
Molybdenum	^{98}Mo	^{72}Ge
Nickel	^{60}Ni	^{72}Ge
Platinum	^{195}Pt	^{193}Ir
Selenium	^{78}Se	^{128}Te
Thallium	^{205}Tl	^{209}Bi
Uranium	^{238}U	^{209}Bi
Vanadium	^{51}V	^{45}Sc
Zinc	^{66}Zn	^{72}Ge

Table S2. ICP-MS instrumental operating conditions.

Component/Parameter	Type/Value/Mode
Nebulizer	Multineb ®
Spray chamber	Glass cyclonic, baffled and cooled
Sampler and Skimmer cones	Ni – tipped
Skimmer cone insert	High matrix
Injector	Quartz, 1.0 mm i.d.
Cool gas flow	14 L·min^{-1}
Auxiliary gas flow	0.8 L·min^{-1}
Nebulizer gas flow	1.04 L·min^{-1}
KED settings	Helium, 4.9 mL·min^{-1}, 3 V
Sample uptake rate	0.40 mL·min^{-1}
RF power	1550 W
Sweeps	20
Replicates	3
Dwell time	50 ms

Table S3. The analytical sequence followed in each batch of samples analysed.

1. Ignite instrument, allow 10 minutes to warm up.
2. Run a Performance Report to check instrument performance.
3. Calibrate using blank and standards.
4. Run Initial Calibration Verification (ICV) standard and Initial Calibration Blank (ICB) to monitor calibration validity after calibration, every ten samples and at the end of the analytical sequence.
5. Run appropriate certified reference materials (CRM) to check for method accuracy (trueness and precision) in compliance with the Quality Control System implemented in the Lab.
6. Run unknown samples.
7. Review results of quality control samples for pass/fail criteria.

Table S4. Polyatomic interferences in ICP-MS

Analyte/mass	Abundance (%)	Interference
^{75}As	100	$^{40}Ar^{35}Cl^+$
^{51}V	99.76	$^{35}Cl^{16}O^+$, $^{38}Ar^{13}C^+$, $^{36}Ar^{15}N^+$
^{59}Co	100	$^{43}Ca^{16}O^+$, $^{42}Ca^{16}O^1H^+$, $^{24}Mg^{35}Cl^+$, $^{36}Ar^{23}Na^+$
^{63}Cu	69.1	$^{40}Ar^{23}Na^+$, $^{47}Ti^{16}O^+$, $^{23}Na^{40}Ca^+$, $^{46}Ca^{16}O^1H^+$
^{65}Cu	30.9	$^{40}Ar^{25}Mg^+$, $^{40}Ca^{16}O^1H^+$,$^{32}S^{33}S^+$
^{55}Mn	100	$^{40}Ar^{14}N^1H^+$, $^{39}K^{16}O^+$, $^{37}Cl^{18}O^+$, $^{40}Ar^{15}N^+$, $^{38}Ar^{17}O^+$, $^{23}Na^{32}S^+$
^{58}Ni	67.77	$^{23}Na^{35}Cl^+$, $^{40}Ar^{18}O^+$, $^{40}Ca^{18}O^+$, $^{40}Ca^{17}O^1H^+$, $^{42}Ca^{16}O^+$, $^{23}Na^{35}Cl^+$
^{60}Ni	26.16	$^{44}Ca^{16}O^+$, $^{23}Na^{37}Cl^+$, $^{43}Ca^{16}O^1H^+$
^{64}Zn	48.89	$^{32}S^{16}O_2^+$, $^{48}Ca^{16}O^+$, $^{32}S_2^+$,$^{34}S^{16}O_2^+$,$^{36}Ar^{14}N_2^+$
^{66}Zn	27.81	$^{34}S^{16}O_2^+$, $^{33}S^{16}O_2{}^1H^+$, $^{32}S^{16}O^{18}O^+$, $^{32}S^{17}O_2^+$,$^{33}S^{16}O^{17}O^+$, $^{32}S^{34}S^+$, $^{33}S_2^+$
^{68}Zn	18.57	$^{36}S^{16}O_2^+$, $^{34}S^{16}O^{18}O^+$, $^{40}Ar^{14}N_2^+$, $^{35}Cl^{16}O^{17}O^+$, $^{34}S_2^+$,$^{36}Ar^{32}S^+$, $^{34}S^{17}O_2^+$, $^{33}S^{17}O^{18}O^+$, $^{32}S^{18}O_2^+$, $^{32}S^{36}S^+$
^{78}Se	23.52	$^{40}Ar^{38}Ar^+$, $^{38}Ar^{40}Ca^+$
^{80}Se	49.82	$^{40}Ar_2^+$, $^{32}S^{16}O_3^+$

Table S5. LOQ and Results for the Reference materials: Seronorm™ - Trace Elements Whole Blood Level I and II.

		Level I				Level II			
Analyte	LOQ (µg·L-1)	Certified Value (µg·L-1)	Certified range (µg·L-1)	Obtained recovery (%)	Obtained recovery range (%)	Certified Value (µg·L-1)	Certified range (µg·L-1)	Obtained recovery (%)	Obtained recovery range (%)
Sb	1.08	2.3	1.9-2.8	104	92-123	21.3	17.0-25.6	105	95-118
As	0.46	2.5	2.0-3.0	104	94-117	11.4	9.1-13.7	110	99-120
Ba	0.84	40.0	32.0-48.0	101	100-102	130.0**	104.0-156.0	105	101-109
Be	0.68	10.2*	8.1-12.2	93	88-98	5	4.0-6.0	94	86-108
Cd	0.15	0.29	0.23-0.35	96	81-117	5	4.0-6.0	90	83-100
Co	0.06	0.2	0.16-0.24	96	85-121	5.1	4.0-6.1	95	88-103
Cs	0.06	10.7*	8.1-12.2	101	100-102	100.7**	80.1-120.1	104	101-109
Cu	1.54	650	520-780	96	86-112	920	830-1000	93	86-108
Pb	0.76	10.3	8.2-12.4	110	95-119	295	264-325	102	93-110
Mn	0.84	16.3	13.0-19.6	105	96-114	22.5	20.7-24.3	105	96-114
Hg	1.0	1.63	1.30-1.96	94	85-116	15	12-18	92	83-107
Mo	0.49	39.6*	31.7-47.5	99	96-102	5.3	4.3-6.4	117	112-121
Ni	0.99	1.4	1.12-1.68	100	86-119	10.1	8.0-12.1	95	86-119
Pt	0.30	10.2*	8.1-12.2	100	99-101	100.2**	80.14-120.2	101	98-104
Se	0.77	67	47-87	114	99-121	91	63-118	113	101-120
Tl	0.04	10.10*	8.08-12.12	101	90-111	10.4	8.3-12.4	108	101-118
U	0.24	10.1*	8.1-12.1	110	102-119	100.1**	80.1-120.1	108	100-121
V	0.48	10.3*	8.2-12.3	101	99-105	4.1	3.3-5.0	103	96-109
Zn	11.6	4500	3600-5400	97	84-123	6200	5000-7500	95	82-117

* denotes sample spiked with 10 µg·L-1 and **denotes sample spiked with 100 µg·L-1. Due to the nature of the CRM, the values provided are approximate or below our LOQ.

Table S6. Elemental mercury analyzer operating conditions.

Parameter	Value
Wavelength	253.7 nm
Drying time	130 s
Drying temperature	200 ºC
Decomposition time	90 s to achieve 650ºC and 90 s maintain at 650ºC
Decomposition temperature	650 ºC
Weight of analysis	100 mg

Table S7. Semiquantification of frequency of consumption of food groups (kg or L/month) procedure

Consumption frequency categories	Conversion factor to nº portions/month
> 6 portions day	180
4-5 portion day	135
2-3 portion day	75
1 portion day	30
5-6 portions week	22
2-4 portions week	12
1 portion week	4
1-3 portions month	2
Never or less than 1 portion month	0.5

For each food consumption variable, participants could select one of nine consumption frequency categories. These frequencies were converted into monthly intake values using predefined conversion factors and then multiplied by the estimated weight/volume of each portion size.
